# Thoracic myelopathy caused by ossification of ligamentum flavum of which fluorosis as an etiology factor

**DOI:** 10.1186/1749-799X-2-6

**Published:** 2007-04-04

**Authors:** Wenbao Wang, Linghua Kong, Heyuan Zhao, Ronghua Dong, Jing Zhou, Yun Lu

The corresponding author, Dr Wenbao Wang, submitted this article [1] to *Journal of Orthopaedic Surgery and Research *shortly after submitting the article to *European Spine Journal*. *European Spine Journal *accepted the article [2] in September 2006, and the article was then also accepted for publication in *Journal of Orthopaedic Surgery and Research *in November 2006. Since it has been brought to the attention of all authors that duplicate submission and publication have taken place the decision has been made to retract the article published in *Journal of Orthopaedic Surgery and Research*. Dr Wenbao Wang accepts full responsibility for this unfortunate situation and is deeply sorry for any inconvenience this may have caused to the editorial staff of *Journal of Orthopaedic Surgery and Research *and its readers.
